# Genetic characteristics of common variable immunodeficiency patients with autoimmunity

**DOI:** 10.3389/fgene.2023.1209988

**Published:** 2023-11-13

**Authors:** Zhihui Liu, Chenyang Lu, Pingying Qing, Ruijuan Cheng, Yujie Li, Xue Guo, Ye Chen, Zhiye Ying, Haopeng Yu, Yi Liu

**Affiliations:** ^1^ Department of Rheumatology and Immunology, West China Hospital, Sichuan University, Chengdu, China; ^2^ Novogene Co. Ltd., Beijing, China; ^3^ West China Biomedical Big Data Center, West China Hospital, Sichuan University, Chengdu, China; ^4^ Med-X Center for Informatics, Sichuan University, Chengdu, China

**Keywords:** common variable immunodeficiency, primary Immunodeficiencies, whole genome sequencing technologies, autoimmunity, oligogenic model

## Abstract

**Background:** The pathogenesis of common variable immunodeficiency disorder (CVID) is complex, especially when combined with autoimmunity. Genetic factors may be potential explanations for this complex situation, and whole genome sequencing (WGS) provide the basis for this potential.

**Methods:** Genetic information of patients with CVID with autoimmunity, together with their first-degree relatives, was collected through WGS. The association between genetic factors and clinical phenotypes was studied using genetic analysis strategies such as sporadic and pedigree.

**Results:** We collected 42 blood samples for WGS (16 CVID patients and 26 first-degree relatives of healthy controls). Through pedigree, sporadic screening strategies and low-frequency deleterious screening of rare diseases, we obtained 9,148 mutation sites, including 8,171 single-nucleotide variants (SNVs) and 977 Insertion-deletions (InDels). Finally, we obtained a total of 28 candidate genes (32 loci), of which the most common mutant was *LRBA.* The most common autoimmunity in the 16 patients was systematic lupus erythematosis. Through KEGG pathway enrichment, we identified the top ten signaling pathways, including “primary immunodeficiency”, “JAK-STAT signaling pathway”, and “T-cell receptor signaling pathway”. We used PyMOL to predict and analyse the three-dimensional protein structures of the *NFKB1, RAG1, TIRAP, NCF2*, and *MYB* genes. In addition, we constructed a PPI network by combining candidate mutants with genes associated with CVID in the OMIM database via the STRING database.

**Conclusion:** The genetic background of CVID includes not only monogenic origins but also oligogenic effects. Our study showed that immunodeficiency and autoimmunity may overlap in genetic backgrounds.

**Clinical Trial Registration:** identifier ChiCTR2100044035

## 1 Introduction

Common variable immunodeficiency disorder (CVID) is the most common primary symptomatic immunodeficiency disease in adults, with a prevalence of 1:50000 to 1:25000 (Sneller et al., 1993; Ameratunga, 2018). The clinical manifestations were highly heterogeneous, mainly showing recurrent infections and hypogammaglobulinemia. Some CVID patients also have autoimmune or inflammatory features, such as progressive interstitial lung disease, autoimmunity, gastrointestinal inflammatory disease, granuloma, lymphoproliferative disorders. The etiology of CVID is mostly unknown, and there is no unified definition. Expansion of the inborn errors of immunity landscape has increasingly revealed that monogenic, polygenic, and epigenetic factors are involved in the onset of CVID. In addition, genotypic and phenotypic overlap between CVID and other immune diseases is also increasing (Bogae et al., 2016; Peng et al., 2023). Therefore, some scholars have proposed that if pathogenic mutations are found (excluding *TNFRSF13B/TACI, TNFRSF13C/BAFFR*, etc.), these patients will be removed from the overall diagnosis of CVID and reclassified as having a CVID-like disease caused by a specific mutation (Ameratunga, 2018; Ameratunga et al., 2018). With the development of sequencing technologies, such as whole exome sequencing and whole genome sequencing (WGS), high-throughput sequencing technology has gradually become an important means for diagnosing and formulating treatment strategies for CVID-like patients (van Schouwenburg et al., 2015; Ameratunga et al., 2018). However, whole exome sequencing has identified pathogenic variants in less than 20% of patients with predominantly antibody deficiency. WGS can detect broader identifications of rare, underlying genetic variants, helping to further understand the contribution of common and rare variants to disease pathogenesis (Edwards et al., 2021). The early clinical manifestations of CVID often lack specificity, leading to delayed diagnosis and poor prognosis. Genetic testing can not only effectively assist diagnosis, but also provide basis for precision medicine (such as targeted immunotherapeutics). WGS is a good choice to solve this problem, which can effectively improve the diagnosis rate.

Current studies on CVID have mainly focused on Caucasians, whereas there are fewer studies on Asian populations (Gutierrez et al., 2018). According to the clinical manifestations, it can be divided into two types, one is mainly characterized by recurrent infections, while the other is often associated with noninfectious diseases, such as autoimmune or inflammatory disease, lymphoproliferative diseases and other complications (Chapel et al., 2008; Resnick et al., 2012). Studies have also shown that patients with the second type of CVID tend to have increased morbidity and poor prognosis (Chapel et al., 2008; Resnick et al., 2012). The cooccurrence of two contradictory situations, immunodeficiency and autoimmunity, seems to be impenetrable. Shared genetic factors may be one reason, but this also adds to some extent to the complexity of CVID diagnosis (Xiao et al., 2014). Currently, the phenotypic spectrum of CVID syndromes or CVID-like disorders is expanding, and an increasing number of cases with autoimmune complications have been reported. Therefore, we reviewed the phenotypes of patients with CVID and autoimmunity enrolled in our hospital, and explored the clinical and genetic characteristics of these patients through WGS.

## 2 Materials and methods

### 2.1 Samples and collection

Patients were recruited into the study through the Department of Rheumatology and Immunology, West China Hospital, Sichuan University. All 16 patients and 26 first-degree healthy controls were Han Chinese. The patients met the ESID diagnostic criteria at the time of enrollment, and also fulfilled the classification criteria of coexisting autoimmunity or inflammation (Ameratunga et al., 2013). The cohort consisted of 4 unrelated patients and 12 core families with a clinical presentation of common variable immunodeficiency disorder (CVID). The baseline data in this study was collected retrospectively from our hospital database. The study protocol was approved by the Ethics Committee of West China Hospital, Sichuan University (ethics application reference number: 2020471). Consent was provided by all patients.

### 2.2 Sequencing and data analysis

WGS was performed on all subjects, including 16 probands and 26 first-degree relatives. Genomic DNA fragments with a size of approximately 350 bp were extracted from peripheral blood mononuclear cells. A DNA library was created by the Illumina paired-end protocol. Whole genome sequencing was performed using the Illumina NovaSeq 6000 platform (Illumina Inc., San Diego, CA, United States) to produce 150-bp paired-end reads with a coverage of at least 4% × 99% of the genome.

The sequenced data were subjected to basecall file conversion and demultiplexing by bcl2fastq software (Illumina) (Supplementary Figure S1). Low-quality reads in the resulting fastq data were eliminated using fastp software (Chen et al., 2018). Then, the reads were aligned to the reference human genome (hs37d5) by the Burrows-Wheeler Aligner (BWA), and duplicate reads were flagged by the sambamba tool (Li and Durbin, 2009; Tarasov et al., 2015).

SAMtools was used to call single-nucleotide variants (SNVs) and insertion deletions (InDels) to generate gVCF (Li et al., 2009). Filter thresholds for raw calls of SNVs and InDels: 1) Read depth >4; 2) root-mean-square mapping quality of covering reads >30; 3) variant quality score >20.

The software used for annotation was ANNOVAR (June 8 2017). The annotation content includes the allele frequencies (AF), deleteriousness and conservation scores from public control datasets, enabling further screening and assessment of variants for possible pathogenicity (Wang et al., 2010).

### 2.3 Rare variant filtering

The rare variant screening was as follows: 1) Only SNVs occurring in exons or splice sites (10 bp splice junctions) were further analysed; 2) synonymous SNVs not related to amino acid changes predicted by dbscSNV or defined by RepeatMasker were also discarded small nonframeshifted (<10 bp) InDels in repetitive regions; 3) Screened for variants with AF less than 0.01 in 1000 genome data (1000 g-all), esp6500siv2-all (http://evs.gs.washington.edu/EVS), gnomAD data (gnomAD-all and gnomAD-EAS) (Auton et al., 2015); 4) Screened variants based on the scores of SIFT, Polyphen, MutationTaster and CADD software. Potentially deleterious variants were retained if more than half of the scores in these four pieces of software supported the deleteriousness of the variant (Karczewski et al., 2020).

We used Control-FREEC (v9.1) and LUMPY (v0.2.13) to detect copy number variable (CNV) and structural variable (SV) mutations, respectively (Boeva et al., 2012; Muona et al., 2015). Performing co-occurrence analysis on the detected CNVs and SVs, with the principle of screening for CNVs and SVs that are common to at least two or more patients and not found in normal individuals. If the reciprocal overlap of two mutations was more than 50%, it was considered the same CNV or SV.

### 2.4 Candidate gene analysis

We collected genes related to PID (including 13 known genes of CVID from the OMIM database) from the reported literature and disease database. PID-related genes were extracted after rare variant filtering analysis for candidate gene screening.

Given the characteristics of the pedigree, homozygous, compound heterozygous and *de novo* variants were considered to be candidate causal variations. Considering the complex inheritance of CVID, oligogenic inheritance patterns were also considered. For sporadic samples, the variants were reserved if only detected in the patient.

The American Academy of Medical genetics and Genomics (ACMG) was used to classify variants (pathogenicity, possible pathogenicity, uncertain significance (VUS), possible benign and benign) (Ri et al., 2015). At the same time, phenolyzer software was used to sort the selected candidate genes according to the disease correlation (Yang et al., 2015). We created protein-protein interactions (PPI) networks by String (version 11.0), focusing preferentially on proteins that interact with proteins encoded by known genes of CVID (Szklarczyk et al., 2019). KEGG was used to enrich candidate genes. In addition, the 3D structures of the mutant proteins from the RCSB protein database were displayed using PyMOL software (https://www.schrodinger.com/products/pymol), and the hydrogen bond changes at the mutation sites of the wild-type and mutant proteins were compared (Goodsell et al., 2020). Sanger sequencing was performed to validate all candidate genes detected by WGS.

## 3 Results

### 3.1 Demographics and clinical parameters

**FIGURE 1:**
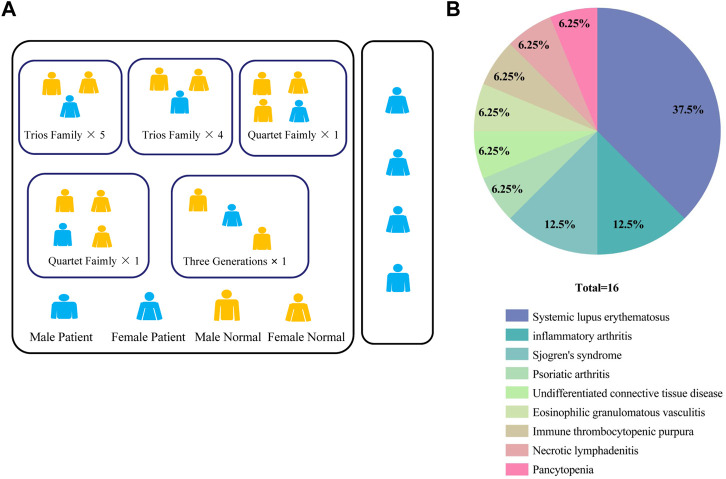
**(A)** Pedigrees in this CVID cohort. **(B)** Percentage of patients with different autoimmune manifestations in the CVID cohort (*n* = 16). CVID, Common variable immunodeficiency disorder; ACMG: The American Academy of Medical Genetics and Genomics.

We enrolled a total of 16 patients with CVID syndrome or CVID-like disorders in the Department of Rheumatology, West China Hospital, Sichuan University from June 2020 to May 2022, with their first-degree relatives as a control group. Finally, 4 sporadic samples, 12 probands and their 26 healthy family samples were collected (Figure 1A). The clinical characteristics of the patients are shown in Supplementary Table S1. Interestingly, in this study, 10/16 (62.5%) patients were complicated with connective tissue diseases, including systemic lupus erythematosus (SLE, 37.5%), Sjogren’s syndrome (12.5%), undifferentiated connective tissue disease (6.25%), and eosinophilic granulomatosis with polyangiitis (6.25%). Others included inflammatory arthritis (12.5%), psoriatic arthritis (6.25%), immune thrombocytopenic purpura (6.25%), necrotizing lymphadenitis (6.25%) and pancytopenia (6.25%) (Figure 1B). Among them, one patient suffered from inflammatory arthritis and myelodysplastic syndrome.

### 3.2 Genetic mutations associated with CVID found by whole genome sequencing

We first performed analysis by whole-genome sequencing of the patients and their first-degree healthy relatives and obtained a total of approximately 4,256 Gb of raw data. The average sequencing depth was 34 x, and the average mapping rate was 99.9%. In our cohort, 26 healthy control samples were selected from the patients’ immediate family members. Therefore, we used two analysis strategies of pedigree and distribution to screen candidate genes.

#### 3.2.1 SNV and InDel

Due to the potentially complex polygenicity and heterogeneity of CVID, we chose mild screening criteria (MAF less than 0.01) and detected a total of 9,578,118 SNVs and 2,758,295 InDels. Through low-frequency deleterious screens, such as frequency, functional region, and deleteriousness, a total of 9′148 mutations were obtained, of which SNV and InDel were 8,171 and 977, respectively. The number of mutations carried by patients was 4,736 SNVs and 528 InDels (Supplementary Tables S2, S3). Meanwhile, to validate our data, we sorted out 551 PID and CVID-related genes (Supplementary Table S3) through the literature and databases (HGMD, OMIM, Phenolyzer), and then screened out the candidate genes related to CVID or PID in our patients.

**FIGURE 2:**
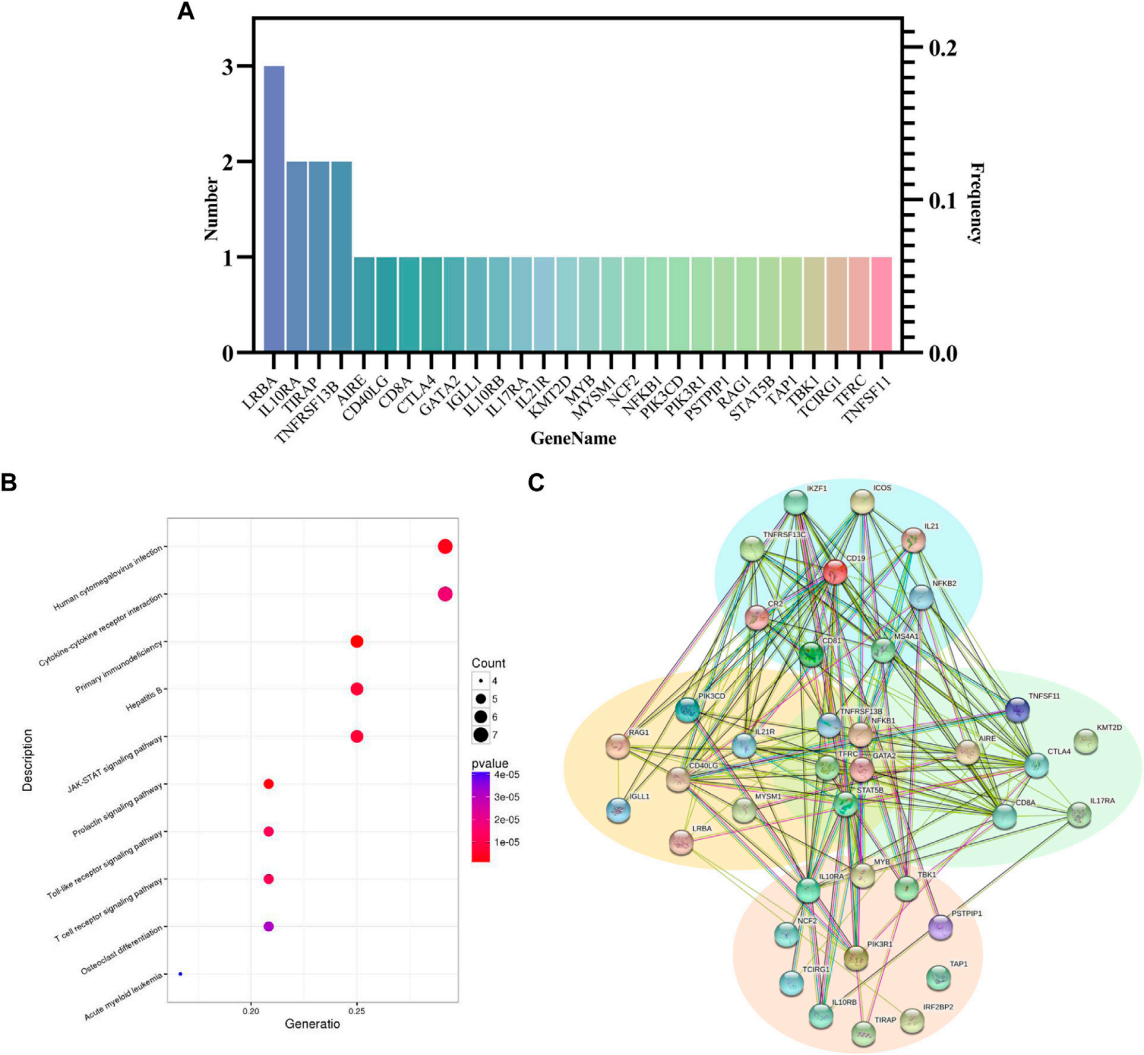
**(A)** Distribution of candidate genes detected by WGS in the CVID cohort. **(B)** KEGG pathway enrichment plots of candidate genes. The top ten pathways are shown in the plot. The bubble size represents gene number enrichment in the pathway, and the color represents the *p*-value. **(C)** Protein-protein interaction networks. The left represents genes related to B cell, the right represents genes related to T-cell, the upper represents genes related to both T and B cells, and the lower represents genes not directly related to either T or B cells.

A total of 9 CVID-related variants were found in our analysis (Supplementary Table S4), the most common of which was LPS-responsive beige-like ankyrin (LRBA), and 3 gene variants (*p. G359D, p. I2744T and p. N2364S*) were found (Figure 2A; Table 1). The three *LRBA* variants were all VUS according to the ACMG classification, which showed a very high level of evolutionary conservation (gerp ++ scores are 5.13, 5.8, and 5.82, respectively). We detected one of the *LRBA* variants (*p.G359D*) with extremely low population frequency as a homozygous, novel mutation that had not been previously reported. Evaluation of this variant by protein damage prediction software (SIFT, Polyphen2_HVAR, Polyphen2_HDIV, MutationTaster) revealed it to be deleterious.

**TABLE 1:** Candidate gene information.

Patient No	Normal name	Heredity	Gene	Function	Het/hom	AAChange	ACMG	Frenquency	Deleterious	gerp++
S1	—	sporadic	*RAG1*	missense	het	p.R582G	VUS	.|	D|B|B|D|12.19	2.13
S2#	—	sporadic	*LRBA*	missense	hom	p.G359D	VUS	.|	D|D|D|D|33	5.13
S3	—	sporadic	*CTLA4*	missense	het	p.P169A	VUS	0.000199681 |0.00004471	D|B|P|D|12.47	<2
	—	sporadic	*TNFRSF13B*	missense	het	p.R84T	VUS	.|0.00002886	D|P|P|N|23	<2
S4	—	sporadic	*AIRE*	splicing	het	c.652+1G>T	VUS	0.000798722 |0.00007992	1.0000,0.906 |-29.5134	3.61
F1	F1_Dad	oligogene	*TNFSF11*	missense	het	p.D317E	VUS	.|	D|P|D|D|	<2
	F1_Mom	oligogene	*PSTPIP1*	missense	het	p.F388L	VUS	0.00239617 |0.00069299	T|B|B|D|11.9	<2
F2	F2_Mom	oligogene	*CD8A*	missense	het	p.R174Q	VUS	.|0.00001222	D|D|D|N|33	3.92
	—	*De novo*	*PIK3R1*	splicing	het	c.336+1G>A	Pathogenic	.|	1.0000,0.938 |-3.9537	5.1
	F2_Mom	oligogene	*TNFRSF13B*	missense	het	p.R84T	VUS	.|0.00002886	D|P|P|N|23	<2
F3	F3_Dad	oligogene	*LRBA*	missense	het	p.I2744T	VUS	0.000199681 |0.00014432	D|P|P|D|24.5	5.8
	F3_Dad	oligogene	*PIK3CD*	missense	het	p.R408C	VUS	0.000599042 |0.00018045	T|B|B|D|18.64	<2
	F3_Mom| F4_Mom	oligogene	*MYSM1*	missense	het	p.P760L	VUS	0.000599042 |0.00053214	D|D|D|D|28.4	5.26
F4	F4_Dad	oligogene	*IL17RA*	missense	het	p.I763V	VUS	0.00279553 |0.00054611	D|B|P|N|19.51	4.55
	F3_Mom| F4_Mom	oligogene	*MYSM1*	missense	het	p.P760L	VUS	0.000599042 |0.00053214	D|D|D|D|28.4	5.26
F5	F5_Dad	oligogene	*TAP1*	stopgain	het	p.E46X	VUS	0.000199681 |0.00010713	.|	2.01
	F5_Mom	oligogene	*IL21R*	splicing	het	c.867 + 10_867 + 28dup	VUS	.|0.00000409	.|	<2
F6	F6_Dad	oligogene	*IL10RA*	missense	het	p.P30L	VUS	.|0.00007214	D|B|P|N|12.45	3.08
	F6_Mom	oligogene	*TIRAP*	missense	het	p.D96N	VUS	0.00139776 |0.00258373	D|D|D|D|29.4	5.41
F7	F7_Dad	oligogene	*TBK1*	missense	het	p.V421L	VUS	0.000199681 |0.00001353	T|B|B|D|20.6	5.27
	F7_Son	oligogene	*IGLL1*	missense	het	p.T146M	VUS	0.00199681 |0.00109133	D|P|P|N|	<2
F8	F8_Dad	oligogene	*LRBA*	missense	het	p.N2364S	VUS	.|0.00000407	T|B|B|D|23.7	5.82
	F8_Mom	oligogene	*IL10RA*	missense	het	p.V233M	VUS	0.0071885 |0.00199517	D|P|D|N|19.48	3.27
	F8_Mom	oligogene	*IL10RB*	missense	het	p.A44V	VUS	0.00119808 |0.00038956	D|P|D|N|22.8	3.47
F9	—	*De novo*	*MYB*	missense	het	p.E132K	VUS	.|	D|D|D|D|31	5.81
F10#	F10-Mom##	recessive	*CD40LG*	missense	hom	p.H125P	Likely Pathogenic	.|	D|P|D|D|25	5.5
F11	—	*De novo*	*GATA2*	stopgain	het	p.R348X	Pathogenic	.|	.|-0.2054	4.05
	F11_Mom	oligogene	*TFRC*	missense	het	p.D70A	VUS	.|0.00004117	T|B|B|D|10.37	<2
	F11_Mom	oligogene	*NFKB1*	missense	het	p.R533H	VUS	0.000199681 |0.00053528	D|D|D|D|29	4.5
	F11_Dad	oligogene	*KMT2D*	missense	het	p.T245S	VUS	.|0.00000455	T|D|D|D|14.18	5.05
	—	*De novo*	*STAT5B*	missense	het	p.N642H	VUS	.|0.00000406	T|D|D|D|24.3	5.33
F12	F12_Mom	oligogene	*NCF2*	missense	het	p.F116S	VUS	.|	D|D|D|D|32	5.56
	F12_Dad	oligogene	*TCIRG1*	missense	het	p.R60W	VUS	0.00159744 |0.00023873	D|D|D|D|33	4.9
	F12_Mom	oligogene	*TIRAP*	missense	het	p.E132K	VUS	0.00159744 |0.00030251	D|D|D|D|29.3	5.67

Note: “#” represents a homozygous mutation; “##” represents that the patient’s mother carries a heterozygous mutation in CD40LG. “/” stands for none. AA Change represents amino acid change. ACMG: The American Academy of medical genetics and genomics. RS: reference snp. VUS: variants of uncertain significance. “." the black dots in column H (RS) represent that this locus is not included in dbSNP, database, “|” (Frenquency) the black dots in column I represent that there is no mutation in the corresponding population database, indicating that the mutation frequency of this site is extremely low. “|” represents 1000 g and GnomAD, database respectively. Deleterious: D: damaging/disease causing, P: possibly damaging, T: tolerated, N: polymorphism, B: benign.

Our analysis identified one patient who was heterozygous for the *p.R582G* variant in *RAG1*, the gene encoding the recombination activating 1 protein. A homozygous variant of CD40LG (p.H125P) was also found in one patient.In addition, four genes related to CVID were screened: *CTLA4 (p. P169A), NFKB1 (p. R533H), IL21R (c.867+10_867+28dup), and TNFRSF13B (p. R84T)*. Three of them were novel mutations, including *CTLA4 (p. P169A), NFKB1 (p. R533H)*, and *IL21R (c.867+10_867+28dup)*.

#### 3.2.2 PID-associated genes

Given that the clinical phenotypes of CVID or CVID-like diseases often overlap with other primary immunodeficiency forms (PIDs), we hypothesized that PID-related genetic variants might exist in the patients in this study. In addition to CVID-related gene variants, we obtained a total of 23 PID-related variants. Our analysis found variants in the *PIK3R1 (c.336+1G>A)* and *PIK3CD (p. R408C)* genes. *PIK3R1 (c.336+1G>A)* is a *de novo*, heterozygous, splice mutation, which is classified as a pathogenic variant according to ACMG. In addition, we also confirmed that there were loss-of-function (LoF) variants in three other genes, *TAP1 (p. E46X), AIRE (c.652+1G>T), and GATA2 (p. R348X)*, respectively. Among them, *GATA2 (p. R348X)* is a stop-gain, heterozygous, pathogenic mutation. The clinical phenotype of the patient with this mutation is very similar to that of CVID. We have already identified different transcript at this mutated site by whole exome sequencing of this patient (Liu et al., 2021).

*MYB* is an oncogene, but a heterozygous, *de novo* variant of this gene (*p. E132K*) emerged in our study. Two variants (*p. P30L, p. V233M*) were found in *IL10RA*, which have been reported to be closely associated with autoinflammatory diseases, such as inflammatory bowel disease (Kara et al., 2019). In addition, we identified heterozygous missense variants in 10 genes, including *CD8A, TNFSF1, PSTPIP1, IL17RA, TCIRG1, TIRAP, MYSM1, IGLL1, KMT2D, and STAT5B*, associated with T and B cells. Through our sorting of PID-related genes, we also found five possible variants of *TBK1, TIRAP, IL10RB, TFRC*, and *NCF2*. Finally, we screened a total of 28 candidate genes (32 loci in total). Meanwhile, 32 candidate loci were validated by Sanger sequencing. Details of all variants are shown in Table 1.

#### 3.2.3 KEGG pathway enrichment analysis of candidate genes

CVID is clinically heterogeneous, with onset in different patients often caused by dysregulation of different signaling pathways. KEGG pathway enrichment was performed based on the candidate genes, and the enrichment results were statistically analysed by R software, with a priority given to immune-related and *p*-value significant pathways. KEGG analysis showed that the top 10 candidate gene-related pathways mainly included “primary immunodeficiency”, “prolactin signaling pathway”, “human cytomegalovirus infection”, “JAK-STAT signaling pathway”, “type B hepatitis”, “T-cell receptor signaling pathway”, “toll-like receptor signaling pathway”, and so on (Supplementary Table S5, Figure 2B). We also performed disease ontology enrichment analysis through the STRING website based on 28 candidate genes. The results showed that the significantly enriched disease pathways included common variable immunodeficiency and autoimmune diseases (Supplementary Table S6).

#### 3.2.4 PPI analysis between CVID-related genes

Protein-protein Interaction networks (PPIs) are composed of proteins that interact with each other to participate in all aspects of life processes, such as biological signal transmission, gene expression regulation, energy and material metabolism, and cell cycle regulation. To further understand the protein-protein interaction, we constructed a visualized PPI network by combining 28 candidate genes and 13 genes associated with CVID in the OMIM database with the STRING database (Supplementary Table S7) As shown in Figures 2A, C total of 36 nodes were included in the PPI network, indicating that these proteins are highly connected with other proteins and interact functionally. In addition, these proteins were divided into four clusters according to whether they were related to T and B lymphocytes. Coding genes for proteins are represented as nodes.

#### 3.2.5 Protein 3D structure prediction

In addition, we performed 3D structure mapping of mutant and wild-type proteins using PyMOL software on candidate mutations for which protein 3D structure data were available in the PDB database, and further analysed whether the amino acid encoded by the mutation has changed hydrogen bonds. After excluding alternative splicing, stopgain, loss of function, incomplete protein structure analysis of missense mutations, and the absence of alterations in the H-bonds before and after mutation, we compared the wild-type and mutant structures of five candidate genes, including *MYB* (AlphaFold), *NFKB1* (AlphaFold), *RAG1* (AlphaFold), *TIRAP* (3UB2), and *NCF2* (1HH8), to elucidate the structural changes in the protein. Since *MYB, NFKB1* and *RAG1* had no analytical results in PDB, we compared them based on the 3D structures predicted by the AlphaFold algorithm. Among them, the mutation occurred in exon 5 of the *MYB* gene (*p. E132K*), and the glutamic acid at position 132 is replaced by lysine, resulting in the disappearance of the hydrogen bond with arginine at position 131 and asparagine at position 136 (distance were 2.9 Å and 2.9 Å, respectively). This variant of *MYB* is haploinsufficient, and the patient carrying the mutant was a 30-year-old female with CVID and myelodysplastic syndrome, who eventually died of septic shock. Although this gene variant (*p. E132K*) has never been reported to be associated with CVID, ACMG classified it as a VUS, with a gerp++ score of 5.81, predicted to be deleterious. Therefore, we speculated that it may be a candidate gene for CVID, but further research is needed to verify its function in pathogenesis.

**FIGURE 3:**
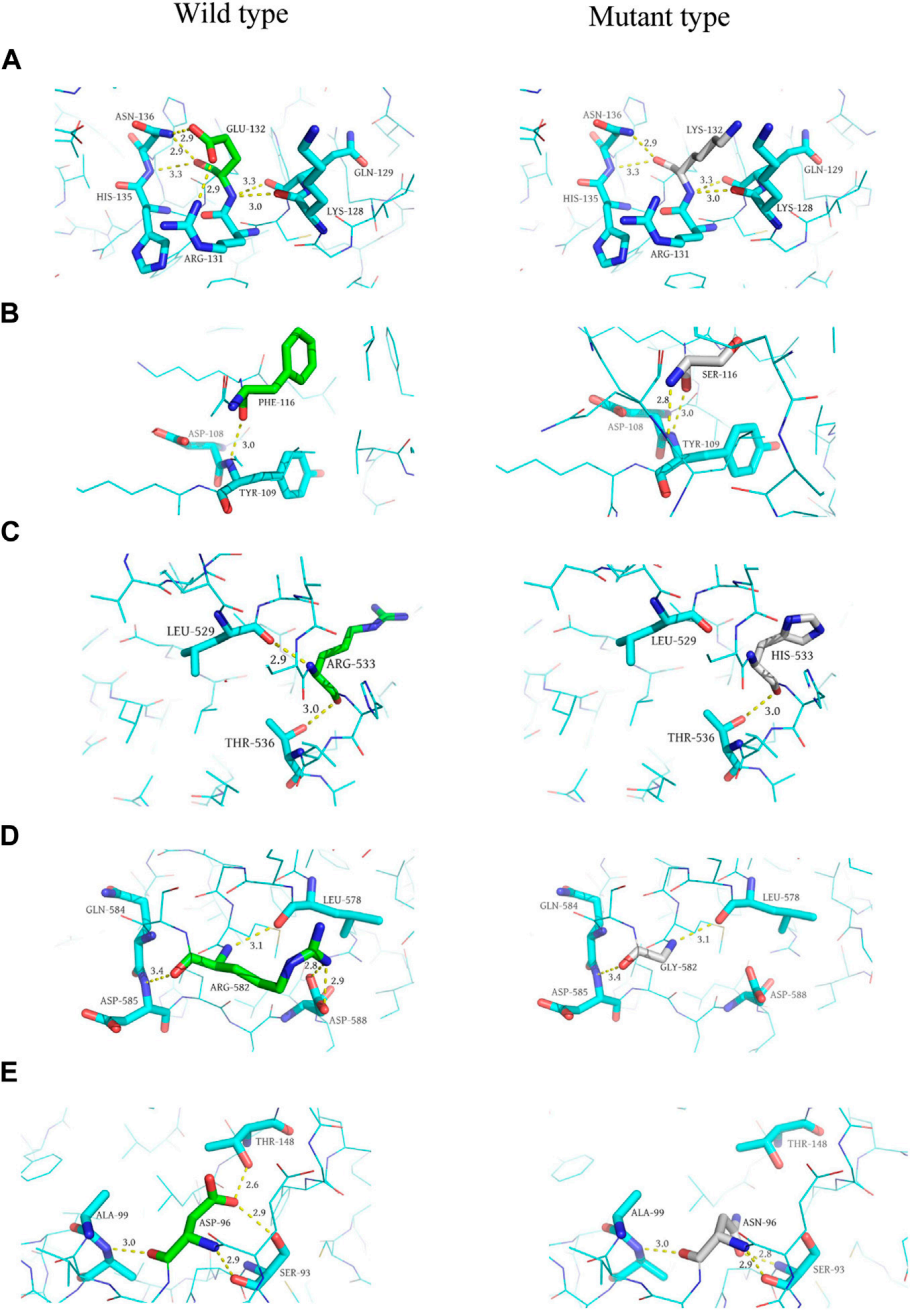
Predicted 3D structure of the protein. **(A, C, E, G, and I)** are the wild-type (left) 3D structures of MYB, NCF2, NFKB1, RAG1, and TIRAP, respectively. **(B, D, F, H, and J)** are the mutant (right) 3D structures of MYB, NCF2, NFKB1, RAG1, and TIRAP, respectively.

The mutation in exon 15 of the gene encoding *NFKB1* (*p. R533H*) results in the disappearance of the 2.9 Å long hydrogen bond with leucine at position 529, and the mutated histidine itself forms a 3.2 Å long hydrogen bond. The mutation located in exon 2 of the *RAG1* gene (*p. R582G*) replaced arginine at position 582 with glycine in the original protein structure, resulting in the disappearance of three hydrogen bonds (2.8 Å, 2.9 Å, 3.4 Å in distance) with aspartic acid at position 588 and glutamine at position 584, respectively. Mutations occurred in exon 4 of the *TIRAP* gene (*p. D96N*), asparagine replaces aspartic acid at position 96, resulting in the disappearance of a long 2.9 Å hydrogen bond with threonine at position 148. Asparagine itself formed an additional hydrogen bond, and the distance of the hydrogen bond with the serine at position 93 changed from 2.9 Å to 2.8 Å. A mutation in exon 3 of the *NCF2* gene (*p. F116S*) resulted in the replacement of phenylalanine at position 116 by serine, forming a 2.8 Å hydrogen bond with leucine at position 114 (Protein structures can be found in Figure 3).

### 3.3 CNV and SV

The results of CNV and SV are shown in Supplementary Tables S8-1, S8-2. Most of the CNVs and SVs shared by more than 2 patients and absent from normal individuals were located in intronic regions. However, no genes related to the clinical phenotype of the patients were found in CNV and SV that cover exons. Therefore, the analysis of pathogenic variants based on CNV and SV did not find the expected candidate variants.

## 4 Discussion

In the present study, WGS was applied to identify genetic mutations in 16 CVID or CVID-like patients with autoimmune features. There were more females than males in our study (10:6), which was lower than that found in a previous study from the rheumatology group of the USIDNET cohort (3.3:1) (Chapel et al., 2008). The results from a study in the United States that enrolled 870 CVID patients revealed that 5.9% had rheumatic disease, with inflammatory arthritis being the most common, and others including SLE, Sjogren’s syndrome, Raynaud’s syndrome, vasculitis, mixed connective tissue disease, and psoriasis (Gutierrez et al., 2018). However, in a recent systematic review, blood autoimmune diseases were the most common in CVID with autoimmunity (Rizvi et al., 2020). Contrary to their findings, the most common connective tissue disease in this study was SLE, which may be related to ethnic differences between the study populations.

In this study, candidate genes of patients with SLE included *TNFSF11, PSTPIP1, TAP1, IL21R, IL10RA, TIRAP, TBK1, IGLL1, LRBA, IL10RB, NCF2, and TCIRG1*, while *LRBA, IL10RA, TIRAP, TAP1, IL10RB and IL21R* have been reported to be associated with the clinical phenotype of SLE (Huang et al., 2004; Webb et al., 2009; Peng et al., 2013; Kara et al., 2019; Liphaus et al., 2020; Tayel et al., 2021). In addition, we found correlations between *TNFSF11, PSTPIP1, TBK1, IGLL1, LRBA, IL10RA, IL10RB* and 13 CVID-related genes recorded in the OMIM database by PPI. Therefore, we hypothesized that there is an overlap between immunodeficiency and autoimmune diseases (such as SLE) in genetic background. Unfortunately, research evidence in this area is scarce and needs further exploration.

LRBA deficiency has been reported to cause a CVID-like clinical phenotype (Costagliola et al., 2021). LRBA deficiency not only shows the characteristics of immunodeficiency, such as repeated infection, but is also often combined with autoimmune cytopenia, lymphoid hyperplasia, rheumatoid arthritis, inflammatory bowel disease and so on. A study in Iran showed that the most frequently mutated gene in CVID patients with an autoimmune phenotype was *LRBA*, which is consistent with our findings (Asgardoon et al., 2020). Three patients with *LRBA* variants in our study had psoriatic arthritis, EGPA, and SLE. The *LRBA* variant carried by patients with psoriatic arthritis is a homozygous, novel mutation (*p. G359D*) that has not been previously reported. LRBA is associated with protein kinase A and can participate in intracellular transport. Moreover, LRBA plays a key role in promoting and maintaining the homeostasis of cytotoxic T lymphocyte antigen-4 (CTLA-4) in Tregs (foxp3+t regulatory cells) and activated T-cells (Burnett et al., 2017; Lo and Abdel-Motal, 2017). Many studies have considered variants in *NFKB1* as a monogenic cause of CVID, and considered heterozygous loss-of-function variants in *NFKB1* to be the most common known monogenic cause of CVID (Fliegauf et al., 2015; Kaustio et al., 2017; Tuijnenburg et al., 2018). However, in our study, there was a 30-year-old male patient with not only *NFKB1* missense mutations but also mutations in the *GATA2*, *TFRC*, *KMT2D*, and *STAT5B genes*. Therefore, the monogenic model cannot account for the complex clinical heterogeneity of CVID, and more than 2 candidate gene loci each were present in at least 11/16 patients in our study. We also demonstrated the interaction between candidate genes by PPI network analysis, including the interaction between LRBA and CTLA-4 protein. (Figure 2C).

KEGG pathway enrichment analysis based on candidate genes showed that the highest ranking was primary immunodeficiency. Our analysis identified mutations in multiple genes involved in this pathway (*AIRE, CD40LG, IGLL1, RAG1, TAP1,* and *TNFRSF13B*). CD40L is mainly expressed on the surface of activated CD4^+^ T cells. It controls the proliferation and differentiation of B cells through interaction with CD40, leading to the conversion of immunoglobulin classes (Bhushan and Covey, 2001). *CD40L* mutations lead to CD40 ligand deficiency, which can lead to a rare X-linked recessive primary immunodeficiency disorder, hyper-IgM syndrome (HIGM), characterized by normal or elevated serum IgM levels and reduced or absent levels of other immunoglobulins (Lee et al., 2005). A homozygous missense mutation in *CD40LG* (*p. H125P*) was detected in a young male patient with a CVID phenotype in our cohort. Although this variant has not been previously reported, a different amino acid change at this site (*c.374A>G:p*. *H125R*) was found in a previous study (Katz et al., 1996). According to ACMG, the mutation is likely pathogenic. He suffered from recurrent pulmonary infections, severe diarrhea, and concomitant *C. malfini* infection. This patient had no family history of immunodeficiency, and his mother was a heterozygous carrier. Finally, according to the WGS analysis, the patient was diagnosed with X-linked hyper-IgM syndrome, and he died of septic shock. Typical HIGM has an early onset with recurrent infections and many complications. The onset of atypical mutations is usually late and can occur in young adulthood. This clinical phenotype is milder, has fewer complications, and is hard to distinguish from CVID (França et al., 2018).

Transcription factors encoded by AIRE play a critical role in the development of central immune tolerance, which promotes negative selection of T cells in the thymus, builds thymic microarchitecture, and induces specific subsets of regulatory T-cells (Bruserud et al., 2016). In our cohort, a middle-aged female patient with this gene mutation (LoF) had a clinical phenotype of CVID with UCTD. Studies have reported that mutations in this gene can lead to autoimmune injuries such as autoimmune polyglandular syndrome type 1 (APS-1) (Eldershaw et al., 2011; Bruserud et al., 2016). TAP plays an important role in the immune system to recognize harmful foreign invasion, while also having a tracking effect on tumor cells (Tabassum et al., 2021). Any defect in the *TAP1* gene results in insufficient tumor tracking, and it may also be associated with some inflammatory or autoimmune diseases (Membrive Jiménez et al., 2021).

Mutations in the *PIK3R1* and *PIK3CD* genes can lead to activated phosphoinositide 3-kinase δ syndrome (APDS), a primary immunodeficiency affecting both humoral and cellular immunity, with some phenotypic similarities to CVID (Bloomfield et al., 2021). In this study, we also reported a 19-year-old male with *PIK3R1* gene mutation. He presented the CVID immunophenotype, accompanied by a significant decrease in CD19^+^CD5^+^ B cells (26/µL, normal range: 175–332/µL) and CD4^+^ T cells (272/µL, normal range: 471–1220/µL). He developed recurrent necrotizing lymphadenitis and died of sudden gastrointestinal bleeding at the age of 20. In pathway analysis, we found that this gene was involved in the JAK-STAT signaling pathway, T-cell receptor signaling pathway and Toll-like receptor signaling pathway. In addition to *PIK3R1*, genes involved in T-cell receptor signaling include *CD40LG, CTLA4, NFKB1*, and *PIK3CD*, and genes involved in Toll-like receptor signaling include *NFKB1, PIK3CD, TBK1*, and *TIRAP*. We found that these genetic variants can be involved in multiple pathways simultaneously, suggesting that they may contribute to B-cell dysfunction by affecting T-B-cell interactions as well as innate immunity.

Previous studies have also reported that *TIRAP (p. D96N, p. E132K)* play a role in innate immunity (George et al., 1950; An et al., 2011). In addition, two patients carried these two missense mutations in the TIRAP gene. Interestingly, both patients exhibited the clinical phenotype of systemic lupus erythematosus. The 3D protein structure of this variant of *TIRAP* (p. *D96N*) was found to be altered, highly conserved (gerp++ score: 5.41), and predicted to be deleterious. The protein encoded by this *TIRAP* gene, is related to the TLR4 signaling pathway and plays an important role not only in innate immunity but also in autoimmunity (Summers et al., 2010; Liu et al., 2014). Studies have found that the TIRAP single nucleotide polymorphism is also a protective factor in systemic lupus erythematosus (Castiblanco et al., 2008).

The pathogenesis of CVID is related to defect in B-cell function and antibody production. In this study, 2/16 cases (12.5%) had the same *TNFRSF13B* mutation (p. *R84T*), which was reported in previous studies on CVID (de Valles-Ibáñez et al., 2018). The mutation of *TNFRSF13B*, which leads to defects in BAFF and April (proliferation inducing ligand) receptor (TACI), is one of the earliest mutations related to CVID (de Valles-Ibáñez et al., 2018). TACI deficiency may cause inefficient class-switching recombination of B cells, which could lead to decreased IgG or IgA levels, consistent with the immunological phenotype of the two patients in our cohort (Castigli et al., 2005; Mohammadi et al., 2009). In addition, *TNFRSF13B/TACI* mutation is also closely related to the development of SLE, but heterozygous mutations are also present in a subset of healthy individuals (Salzer et al., 2007). Therefore, scholars believe that *TNFRSF13B* mutations have no determinant role in CVID and can be considered modifier genes or risk factors for CVID (de Valles-Ibáñez et al., 2018).

The genetic background of CVID is very complex, and there is no definite and uniform genetic diagnosis model. To solve this query, WGS could detect a comprehensive range of variation sites, and can also detect splicing sites that are far away from the exonic region and affect variable splicing [e.g., *IL21R (c.867+10_867+28dup)*]. A study of Oxford University Hospital by WGS combined with RNA-sequencing in patients with CVID, the results of which identified the polygenicity of CVID and suggested a number of individual specific mutations (van Schouwenburg et al., 2015). In addition, there are mainly focused on the SNPs of CVID, the evidence is less about the genetic susceptibility of CNVs and SV. Keller M et al. analyzed the association between CNVs burden and CVID, and found that the increase in CNVs burden in CVID was static and intrinsic (Keller et al., 2014). And, Orange JS et al. found that CNVs had multiple novel susceptibility loci for CVID in their study, providing new insights into the genetic variation of CVID (Orange et al., 2011). In this study, we also attempted to analyze the association of CNVs and SV with genetic susceptibility to CVID, but the results were regrettable.

However, there are still some limitations. First, our current sample size is relatively small, and we need to expand the sample size in the future to explore more reliable genetic features. Second, this study is a single-center study. Third, if a meta-analysis of GWAS in different regions can be performed, the conclusions can be made more convincing. This is also what we originally intended to do but did not achieve. Because there are few WGS studies on CVID, data from other studies were not available.

In conclusion, this study provided genetic variants in Chinese adults with autoimmune phenotypes with CVID or CVID-like syndrome. Monogenic and polygenic patterns may participate in the pathogenesis of CVID, and there may be a shared genetic background between immunodeficiency and autoimmunity.

## Data Availability

The raw sequence data reported in this paper have been deposited in the Genome Sequence Archive (Chen et al., 2021) in National Genomics Data Center (Xue et al., 2022), China National Center for Bioinformation/Beijing Institute of Genomics, Chinese Academy of Sciences (GSA-Human: HRA005867) that are publicly accessible at https://ngdc.cncb.ac.cn/gsa-human.

## Abbreviations

CVID, common variable immunodeficiency disorder; InDels, insertion-deletions; KEGG, kyoto encyclopedia of genes and genomes; OMIM, online mendelian inheritance in man; SNVs, single-nucleotide variants; STRING, search tool for recurring instances of neighbouring genes; WGS, whole genome sequencing.
