# Supplementary material for: Genetic characteristics of common variable immunodeficiency patients with autoimmunity
Source: Front Genet. 2023 Nov 13;14:1209988. doi: 10.3389/fgene.2023.1209988 (PMC10679925; doi:10.3389/fgene.2023.1209988)
Supplement: Supplementary file 1 [file Presentation1.PPTX]

## Slide 1
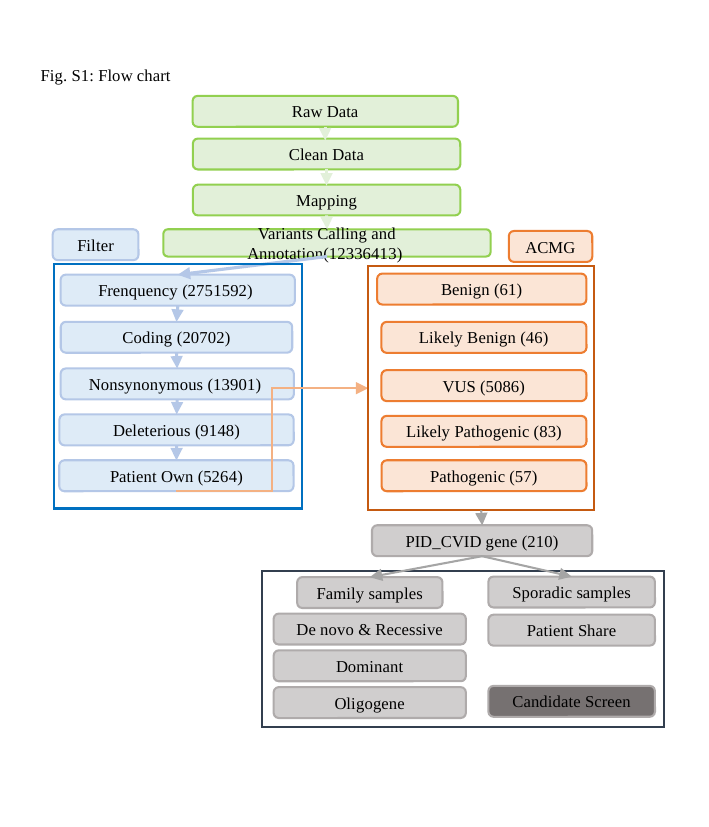

Fig. S1: Flow chart
Raw Data
Clean Data
Mapping
Variants Calling and Annotation(12336413)
Frenquency (2751592)
Coding (20702)
Nonsynonymous (13901)
Benign (61)
Likely Benign (46)
Deleterious (9148)
Patient Own (5264)
VUS (5086)
Likely Pathogenic (83)
Pathogenic (57)
PID_CVID gene (210)
Filter
ACMG
Sporadic samples
Family samples
De novo & Recessive
Patient Share
Dominant
Oligogene
Candidate Screen
